# Supplementary material for: Machine learning-based investigation of the cancer protein secretory pathway
Source: PLoS Comput Biol. 2021 Apr 5;17(4):e1008898. doi: 10.1371/journal.pcbi.1008898 (PMC8049480; doi:10.1371/journal.pcbi.1008898)
Supplement: S1 Text — (DOCX) [file pcbi.1008898.s001.docx]

**S1 Text. Regression analysis of tumor stages.**

Tumor stages were analyzed in the main text as pairs to allow the use of the same binary classification scheme and ML algorithms throughout the study. Here we explore the use of a regression formulation, where tumor stages I–IV were assigned numerical values 1–4, respectively. We note that this quantitative scale implies an equal, linear change in disease severity with increasing tumor stage, which is a heavy oversimplification of tumor pathophysiology. Furthermore, the ML algorithms and scoring metric (ROC AUC) that were used for the binary classification analyses are not compatible with the regression approach. We therefore used the “regressor” form of the ML classification algorithms: random forest regressor, extra trees regressor, lasso regression, ridge regression, adaptive boosting regressor, extreme gradient boosted trees regressor, and support vector regression. Linear discriminant analysis (LDA) was excluded as it cannot be applied in a regression framework. The negative mean squared error (MSE) was used in place of ROC AUC to quantify relative model performance. Finally, only cancer types with at least 10 samples in all four cancer stages (I–IV) were included in the analysis, corresponding to a total of 10 cancer types.

The regression-based analysis of tumor stages in the 10 different cancer types did not result in any high-scoring gene outliers when considering the average ML score across all cancer types (S8A and S8B Fig). KIF20A was among the top-scoring genes on average, which is consistent with the results of the classification approach, though the difference in gene scores was modest and likely not robust. Inspection of the regressor performance scores (negative MSE, S8C-E Fig) showed little difference among different cancer types except for BRCA, which exhibited a modestly improved score on average (S8D Fig). In terms of ML algorithm, ridge regression and support vector regression exhibited the lowest scores on average, whereas the other algorithms were approximately equal (S8E Fig).

The small difference in consensus ML gene scores among even the top genes when averaged across all cancer types suggested that the high-scoring genes were likely more cancer-type specific. A heatmap of the top 3 scoring genes within each cancer type (S8F Fig) illustrates that this is indeed the case, where the top pan-cancer average genes such as KIF20A and B4GALT3 only score highly in 2–3 cancer types out of 10. The two cancer types for which KIF20A received the highest ML scores were the renal carcinomas (KIRP and KIRC), which is consistent with the more gradual, linear expression increase observed among tumor stages in those cancer types (Fig 5C).
