# Supplementary figures and images for: Machine learning-based investigation of the cancer protein secretory pathway
Source: PLoS Comput Biol. 2021 Apr 5;17(4):e1008898. doi: 10.1371/journal.pcbi.1008898 (PMC8049480; doi:10.1371/journal.pcbi.1008898)

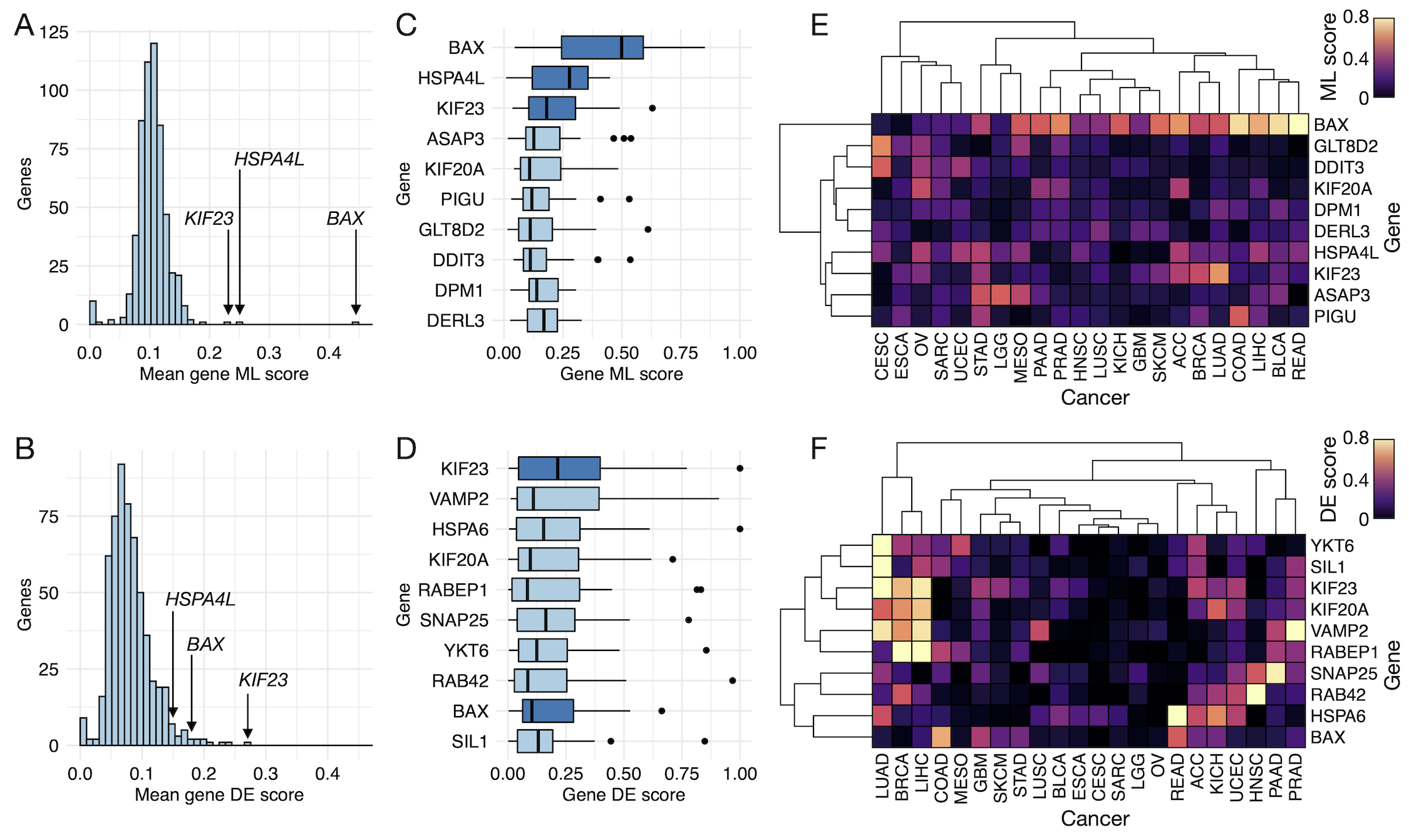

Supplement: S1 Fig — Histogram of (A) mean ML gene scores and (B) mean DE gene scores across all available cancer types, where the PSP genes known to be directly regulated by p53 are labeled. Boxplots of (C) consensus ML gene scores and (D) DE gene scores for the top 10 scoring genes on average. Clustered heatmaps showing the (E) consensus ML gene scores and (F) DE gene scores for individual cancers for the top 10 scoring genes on average. (TIF) [file pcbi.1008898.s002.tif]

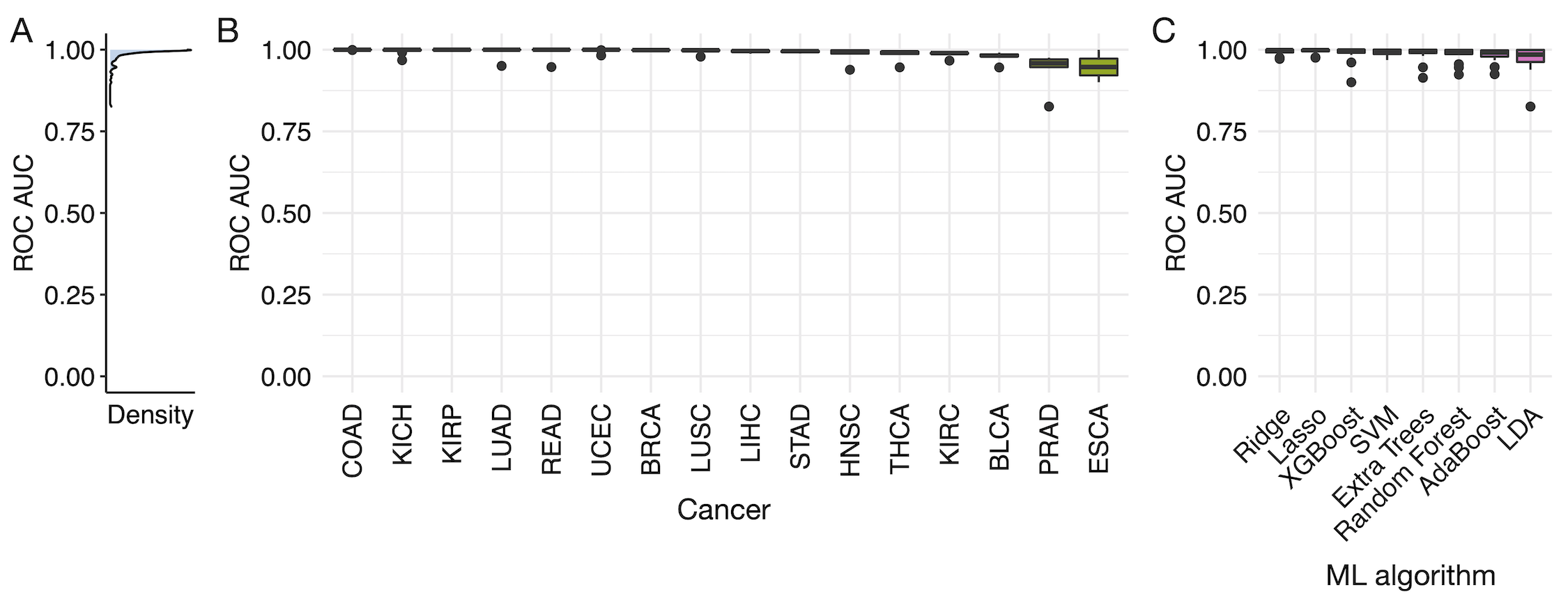

Supplement: S2 Fig — (A) Density histogram of all ROC AUC values across different cancer types and ML algorithms. Boxplots showing the ROC AUC values grouped by (B) cancer type or (C) ML algorithm. (TIF) [file pcbi.1008898.s003.tif]

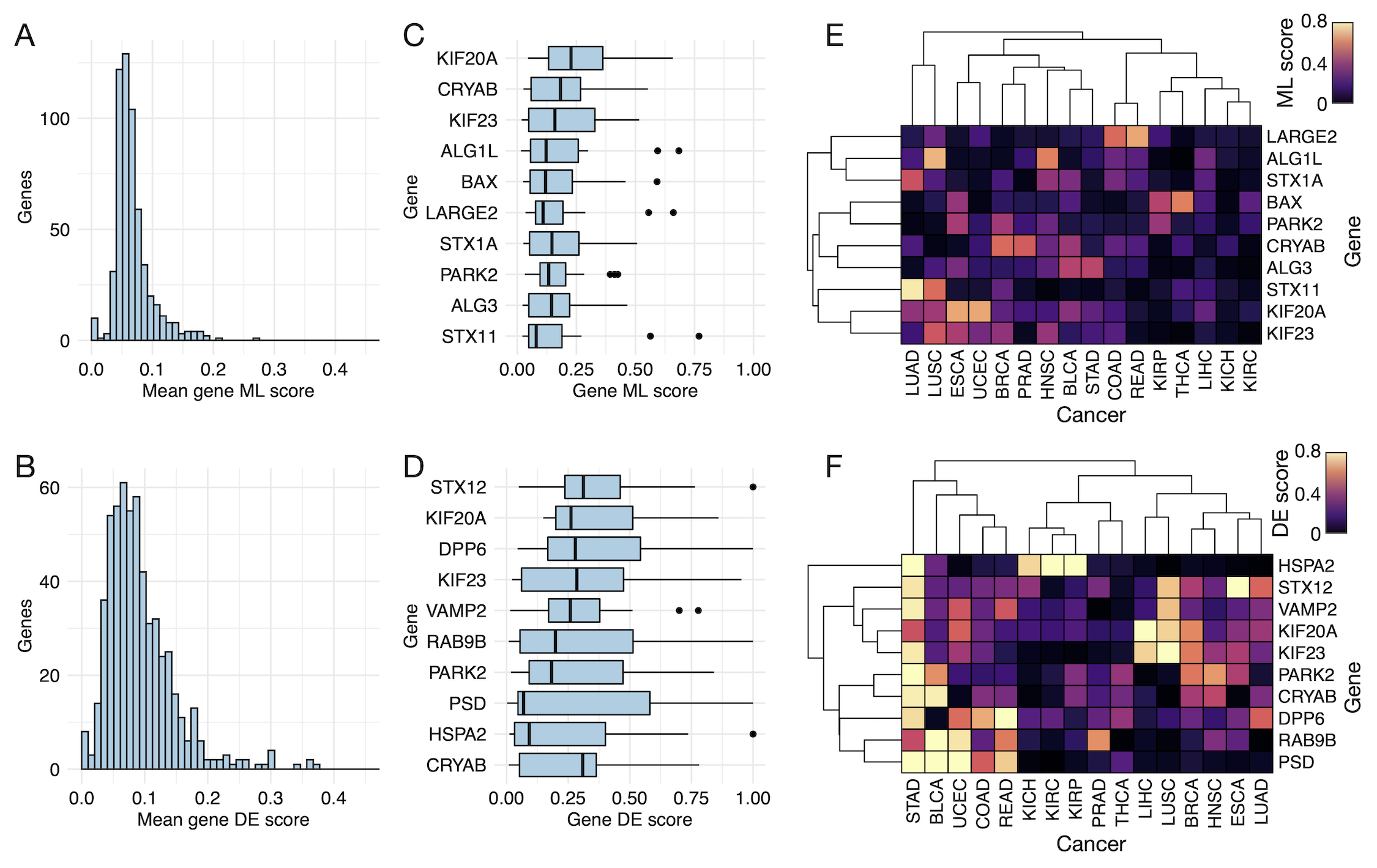

Supplement: S3 Fig — Histogram of (A) mean ML gene scores and (B) mean DE gene scores across all available cancer types. Boxplots of (C) consensus ML gene scores and (D) DE gene scores for the top 10 scoring genes on average. Clustered heatmaps showing the (E) consensus ML gene scores and (F) DE gene scores for individual cancers for the top 10 scoring genes on average. (TIF) [file pcbi.1008898.s004.tif]

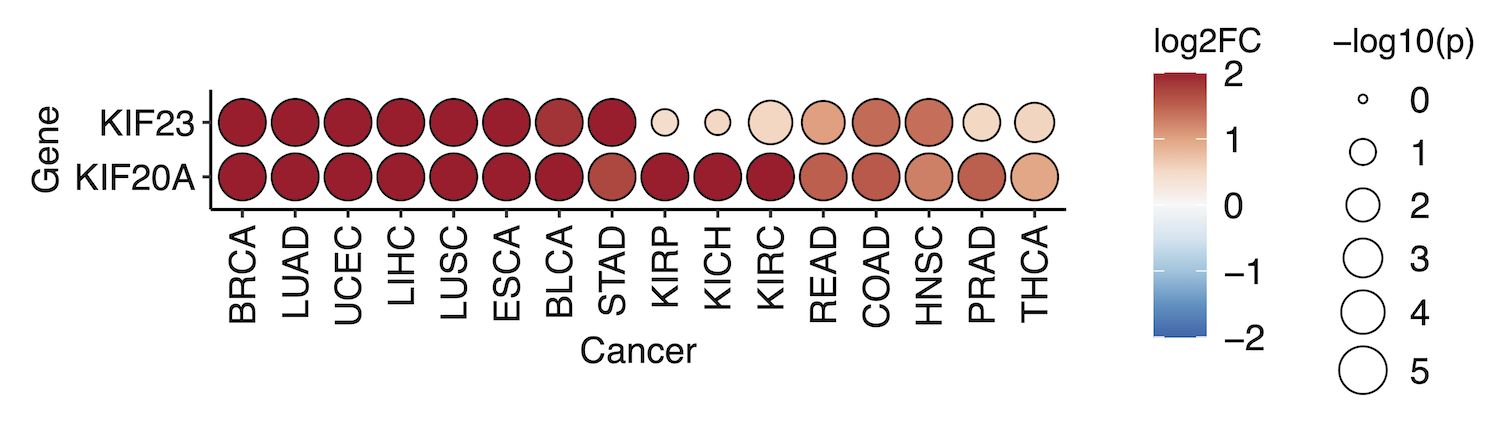

Supplement: S4 Fig — Color indicates fold-change magnitude and direction, whereas circle size indicates significance (FDR-adjusted p-value). (TIF) [file pcbi.1008898.s005.tif]

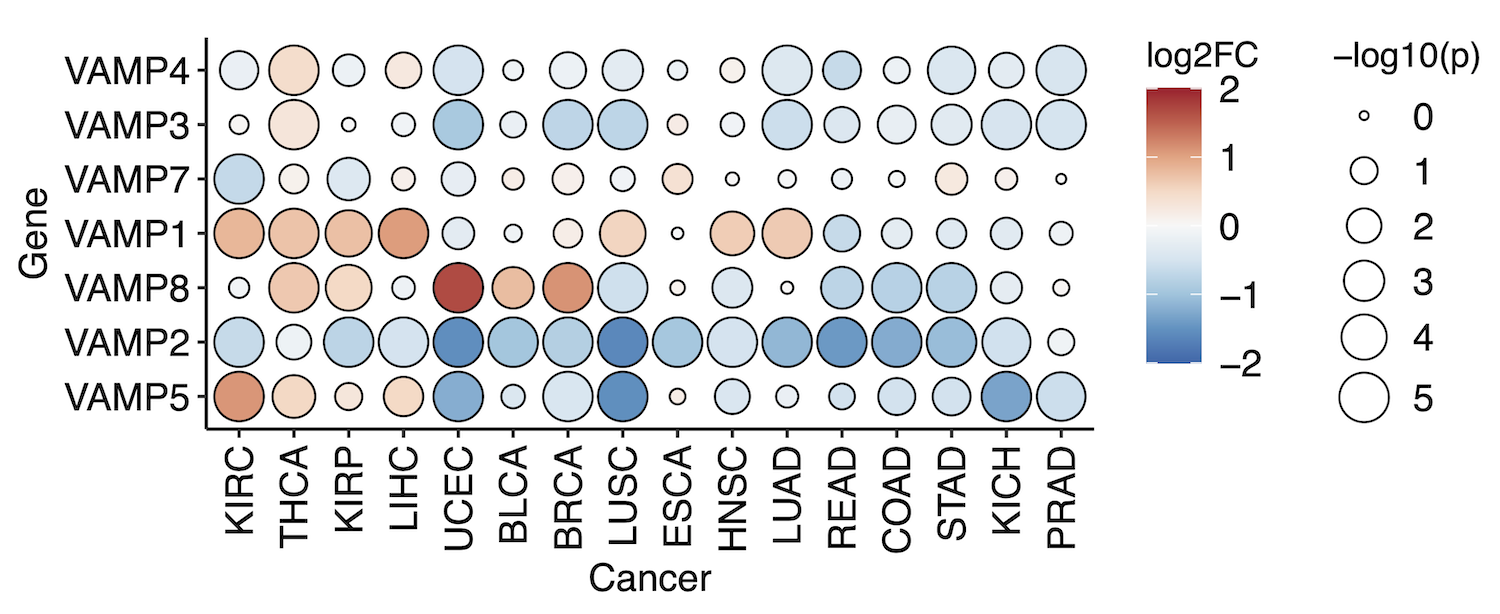

Supplement: S5 Fig — Color indicates fold-change magnitude and direction, whereas circle size indicates significance (FDR-adjusted p-value). (TIF) [file pcbi.1008898.s006.tif]

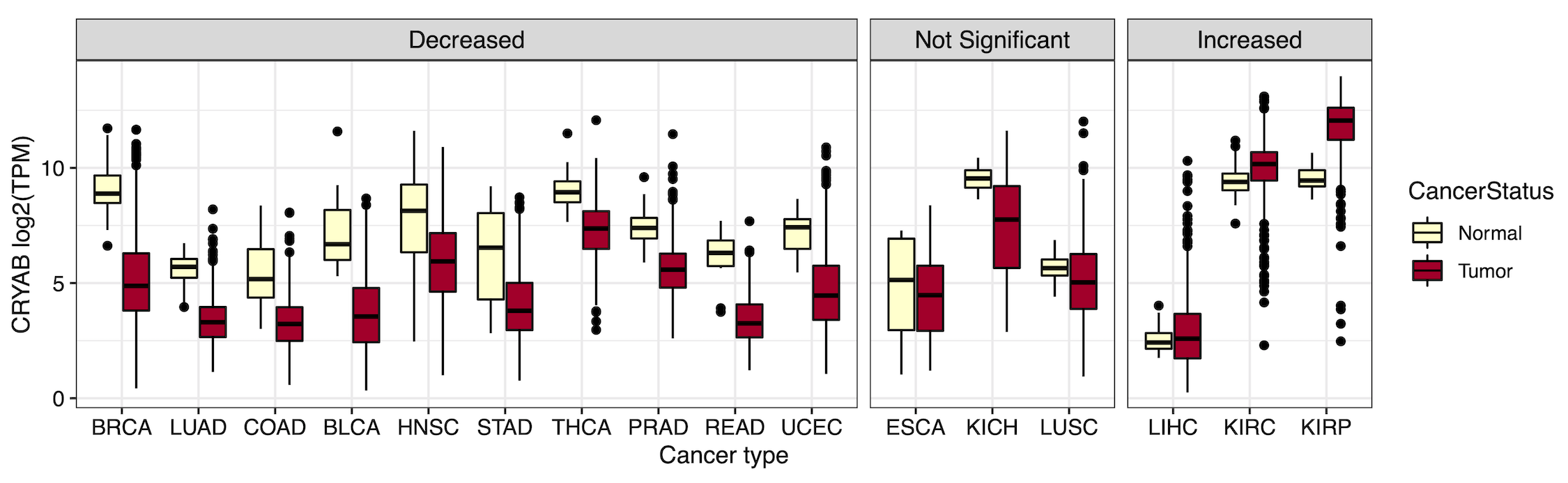

Supplement: S6 Fig — Cancer types are grouped according to whether CRYAB significantly (FDR-adjusted p-value < 0.01) changed in expression between normal and tumor, and whether that change was a decrease or increase. (TIF) [file pcbi.1008898.s007.tif]

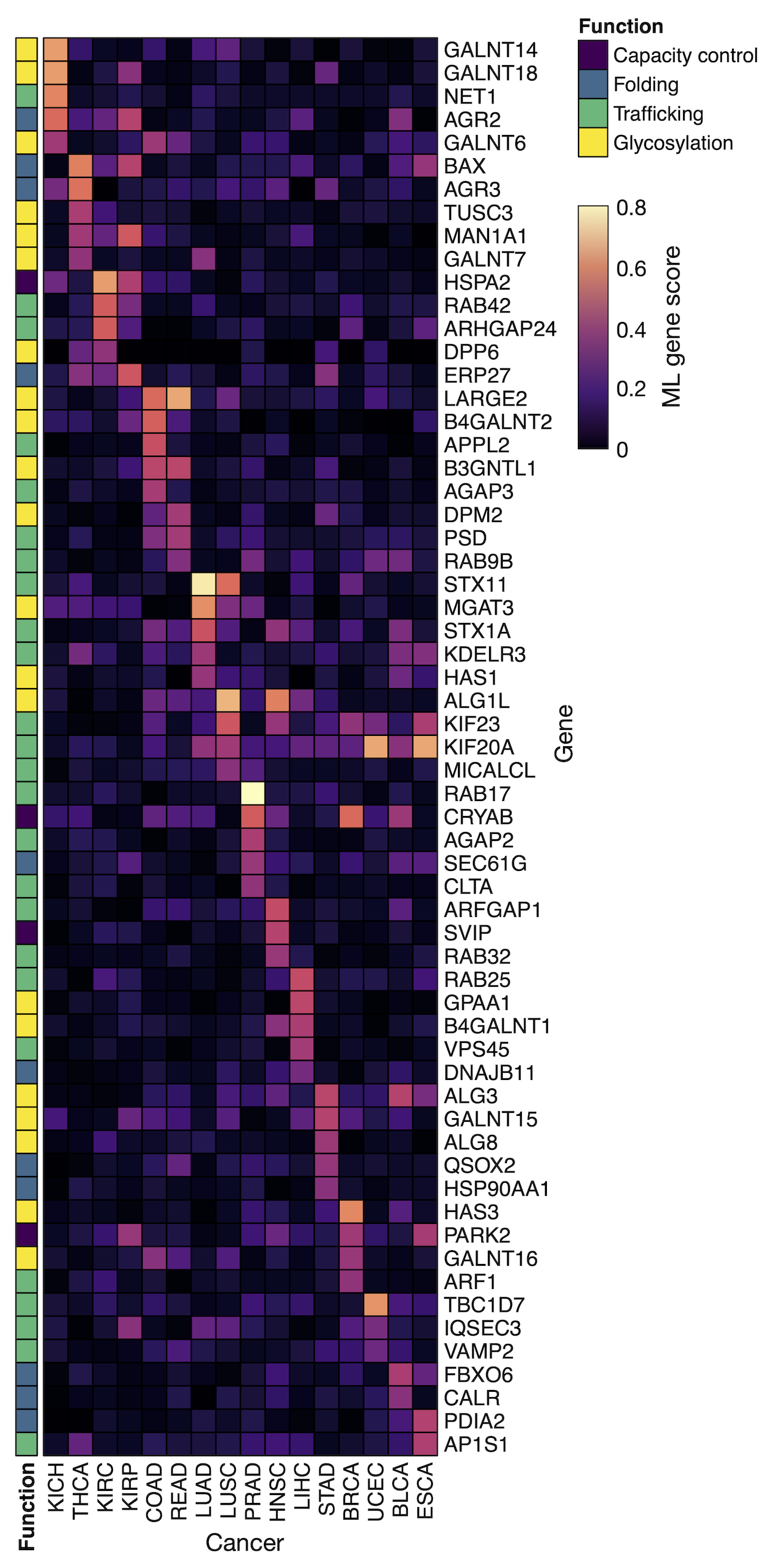

Supplement: S7 Fig — The heatmap includes all available cancer types and the top 5 scoring genes of each type. For visual aid, rows and columns were clustered such that high-scoring genes for each cancer tend to lie along or near the diagonal. The colorbar to the left of the heatmap indicates the function associated with each gene. (TIF) [file pcbi.1008898.s008.tif]

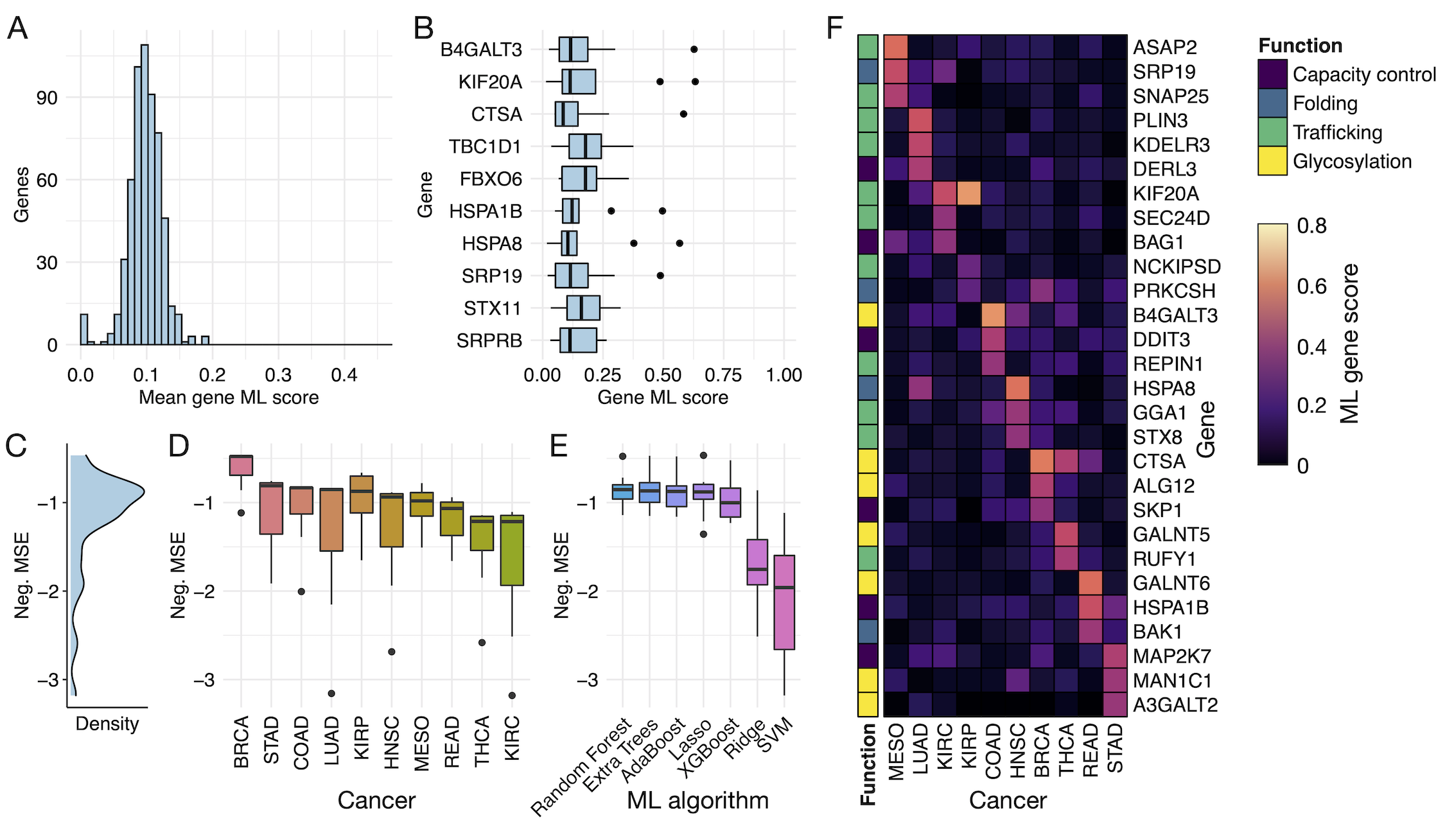

Supplement: S8 Fig — (A) Histogram of mean ML gene scores across all cancer types. (B) Consensus ML gene scores for the top 10 scoring genes on average. (C) Density histogram of negative mean squared error (Neg. MSE) values across different cancer types and ML algorithms. Boxplots showing the negative MSE values grouped by (D) cancer type or (E) ML algorithm. (F) Heatmap of consensus ML gene scores, showing the top 5 scoring genes of each cancer type. The colorbar to the left of the heatmap indicates the function associated with each gene. (TIF) [file pcbi.1008898.s009.tif]

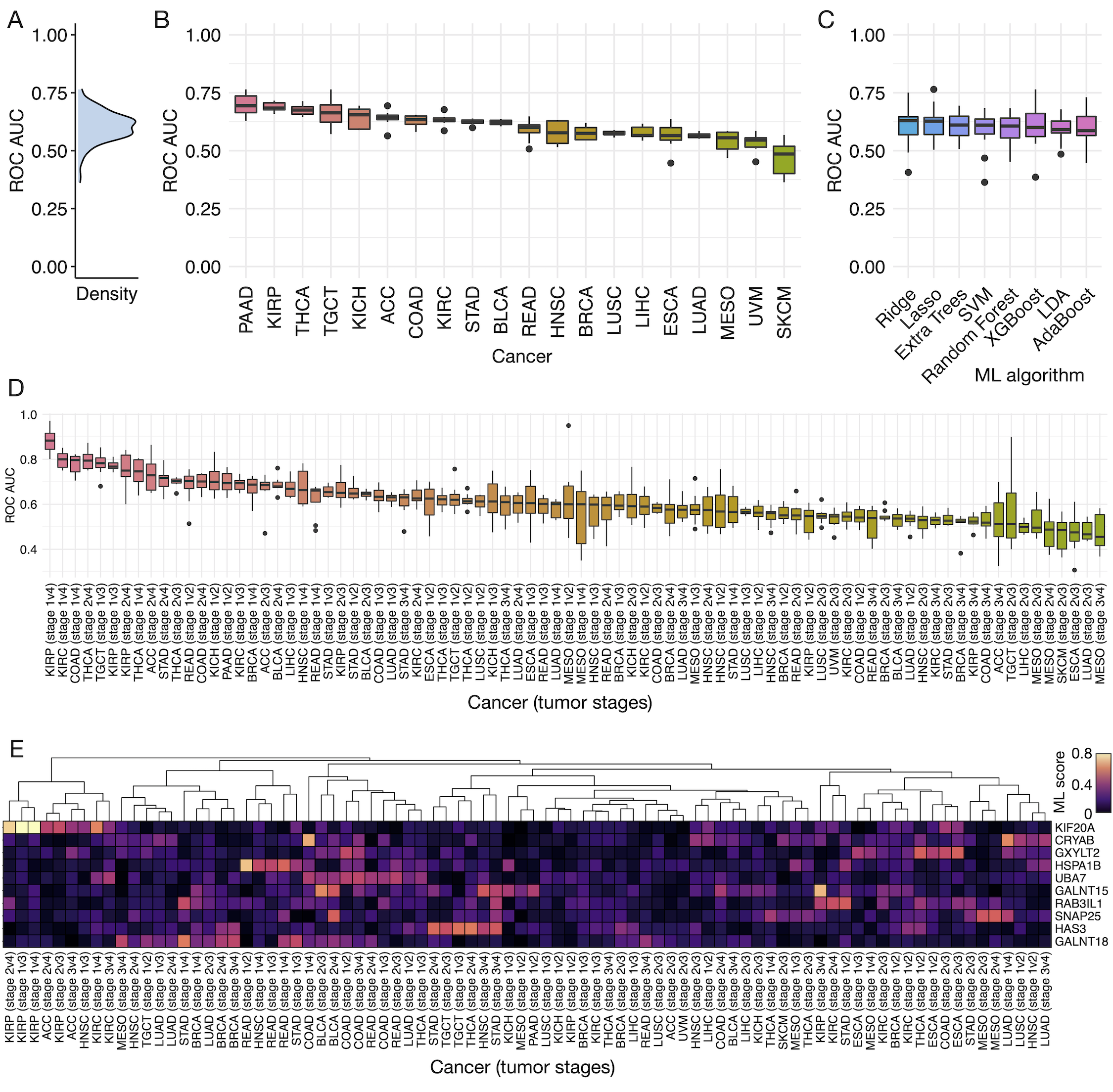

Supplement: S9 Fig — (A) Density histogram of all ROC AUC values across different cancer types and ML algorithms. Boxplots showing the ROC AUC values grouped by (B) cancer type, (C) ML algorithm, or (D) all possible tumor stage pairs. (E) Heatmap showing the consensus ML gene scores for the top 10 scoring genes on average, including all possible tumor stage pairs. (TIF) [file pcbi.1008898.s010.tif]

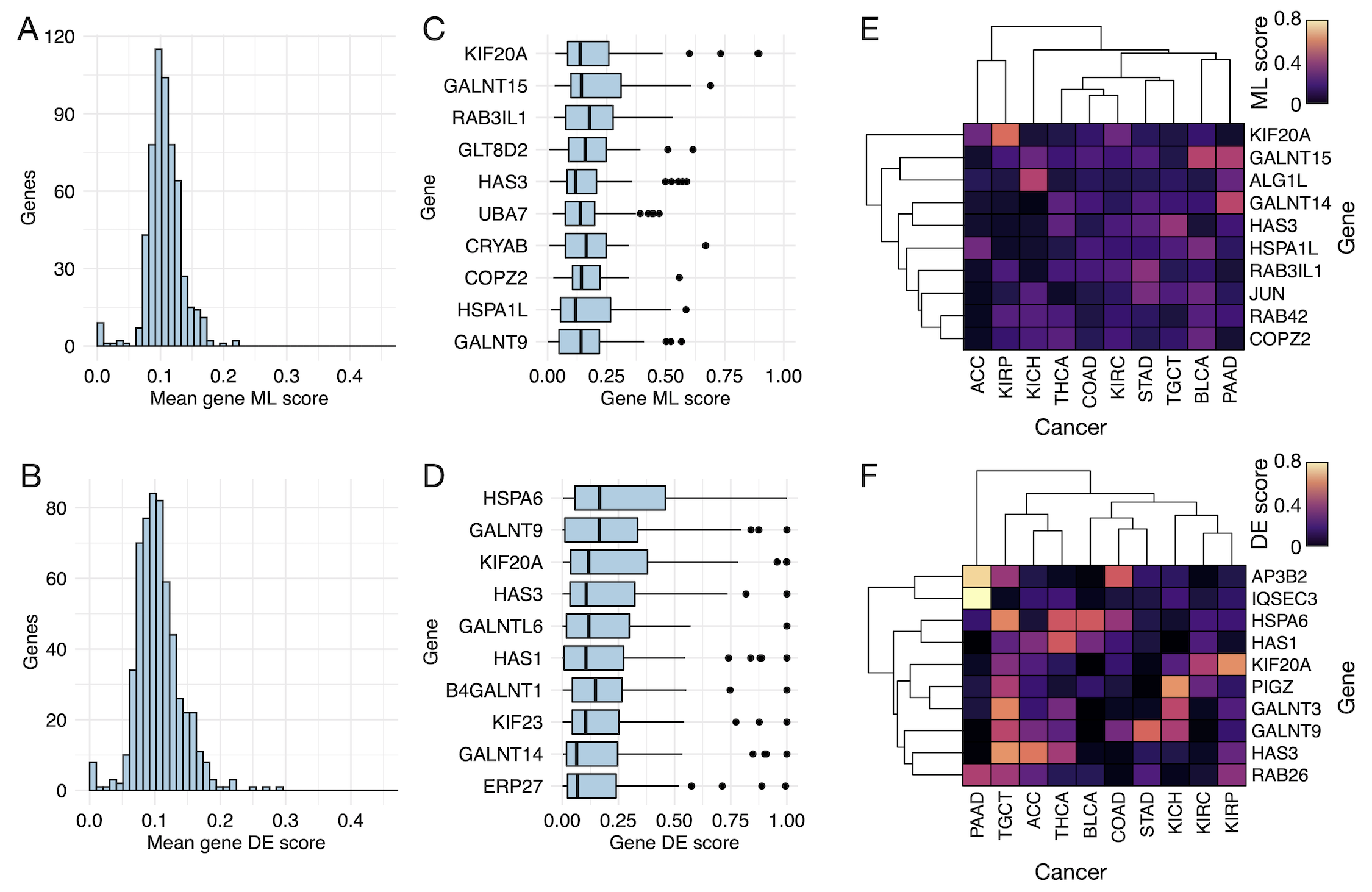

Supplement: S10 Fig — Histogram of (A) mean ML gene scores and (B) mean DE gene scores across the 10 cancer types with the highest average ROC AUC values. Boxplots of (C) consensus ML gene scores and (D) DE gene scores for the top 10 scoring genes on average. Clustered heatmaps showing the (E) consensus ML gene scores and (F) DE gene scores for individual cancers for the top 10 scoring genes on average. (TIF) [file pcbi.1008898.s011.tif]

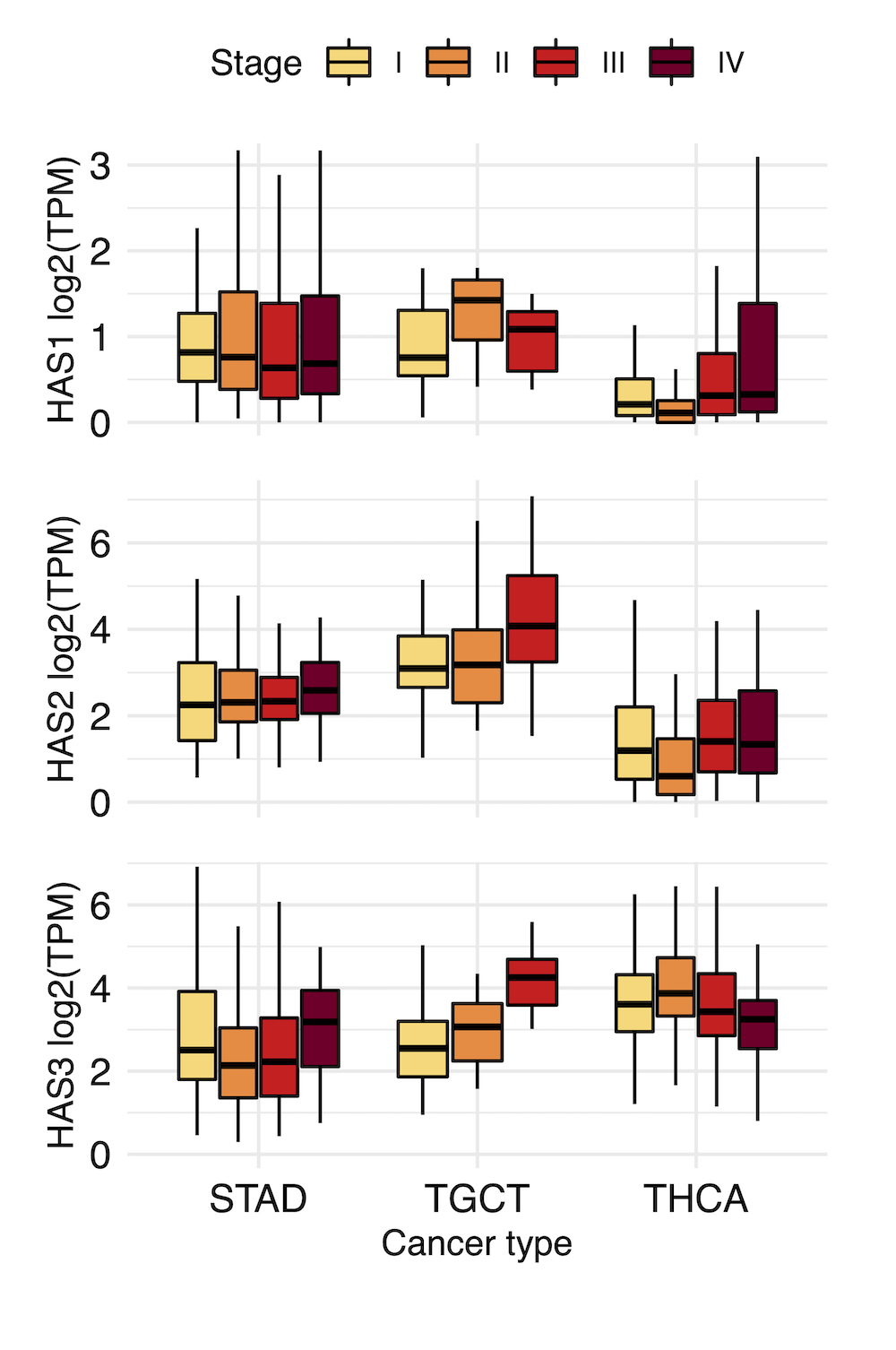

Supplement: S11 Fig — Boxplots present the expression of HAS1, HAS1, and HAS3 in different tumor stages of STAD, TGCT, and THCA cancer types. Note that the stage IV expression levels are lacking for TGCT because no stage IV samples were available for this cancer type. (TIF) [file pcbi.1008898.s012.tif]
