# Supplementary material for: Machine learning-based investigation of the cancer protein secretory pathway
Source: PLoS Comput Biol. 2021 Apr 5;17(4):e1008898. doi: 10.1371/journal.pcbi.1008898 (PMC8049480; doi:10.1371/journal.pcbi.1008898)
Supplement: S5 Table — (DOCX) [file pcbi.1008898.s017.docx]

**S5 Table.** Evaluation of potential p53 regulatory targets identified by the STRING database.

| **Gene** | **STRING Experimental interaction score** | **Confirmed target** | **Refs.** |
| --- | --- | --- | --- |
| BAX | 0.578 | Yes | [1] |
| HSPA1L | 0.555 | No |  |
| HSPA1B | 0.535 | No |  |
| BCL2 | 0.525 | Conflicted | [2–4] |
| UBE2E1 | 0.522 | No |  |
| PARK2 | 0.472 | Conflicted | [5,6] |
| BAK1 | 0.461 | Yes | [7] |
| HSP90AA1 | 0.432 | No |  |
| RAD23A | 0.412 | No |  |
| HSP90AB1 | 0.410 | No |  |
| HSPA8 | 0.406 | No |  |
| HSP90B1 | 0.401 | No |  |

**References**

1. Fischer M. Census and evaluation of p53 target genes. Oncogene. 2017;36: 3943–3956.

2. Budhram-Mahadeo V, Morris PJ, Smith MD, Midgley CA, Boxer LM, Latchman DS. p53 suppresses the activation of the Bcl-2 promoter by the Brn-3a POU family transcription factor. J Biol Chem. 1999;274: 15237–15244.

3. Wu Y, Mehew JW, Heckman CA, Arcinas M, Boxer LM. Negative regulation of bcl-2 expression by p53 in hematopoietic cells. Oncogene. 2001;20: 240–251.

4. Fischer M, Steiner L, Engeland K. The transcription factor p53: not a repressor, solely an activator. Cell Cycle. 2014;13: 3037–3058.

5. Zhang C, Lin M, Wu R, Wang X, Yang B, Levine AJ, et al. Parkin, a p53 target gene, mediates the role of p53 in glucose metabolism and the Warburg effect. Proc Natl Acad Sci U S A. 2011;108: 16259–16264.

6. Fischer M, Grossmann P, Padi M, DeCaprio JA. Integration of TP53, DREAM, MMB-FOXM1 and RB-E2F target gene analyses identifies cell cycle gene regulatory networks. Nucleic Acids Res. 2016;44: 6070–6086.

7. Graupner V, Alexander E, Overkamp T, Rothfuss O, De Laurenzi V, Gillissen BF, et al. Differential regulation of the proapoptotic multidomain protein Bak by p53 and p73 at the promoter level. Cell Death Differ. 2011;18: 1130–1139.
